# Pathways from economic hardship to couple conflict by socioeconomic status during COVID‐19 in Korea

**DOI:** 10.1111/fare.12771

**Published:** 2022-09-09

**Authors:** Jaerim Lee, Jaeeon Yoo, Meejung Chin, Seohee Son, Miai Sung, Young Eun Chang

**Affiliations:** ^1^ Department of Child Development and Family Studies and the Research Institute of Human Ecology Seoul National University Seoul Republic of Korea; ^2^ Department of Social Welfare Gachon University Seongnam Republic of Korea; ^3^ Department of Family & Resource Management Sookmyung Women's University Seoul Republic of Korea; ^4^ Division of Human Ecology Korea National Open University Seoul Republic of Korea; ^5^ School of Social Welfare Chung‐Ang University Seoul Republic of Korea

**Keywords:** COVID‐19 pandemic, economic stress, Korean families, marital relationship

## Abstract

**Objective:**

The purpose of this study was to examine the direct and indirect relationships among economic hardship, economic strain, emotional stress, and couple conflict for married Koreans during the COVID‐19 pandemic. In particular, we investigated whether these pathways were different between lower and higher socioeconomic status (SES) groups.

**Background:**

Due to the global economic downturn brought on by COVID‐19, many couples experienced economic hardship including increased household debt, job loss, and reduced work hours. This context provides a valuable opportunity to test the family stress model (FSM) of romantic relationships, which explains the indirect pathways from economic hardship to couple‐level outcomes.

**Method:**

We collected the data using an online survey in May 2020, when the Seoul metropolitan area experienced the first surge of COVID‐19 cases. The sample came from 605 married Korean adults (282 women, 323 men) and was analyzed using multigroup path analysis.

**Results:**

Among the three markers of economic hardship, increased household debt had a stronger association with couple conflict for *lower* SES respondents directly and indirectly through elevated economic strain and emotional distress. The total effects of job loss and reduced work hours on more frequent couple conflict were stronger for the *higher* SES group.

**Conclusion:**

The process from the three markers of economic hardship to couple conflict was different depending on socioeconomic resources.

**Implications:**

Family practitioners need to consider SES variations and to work with financial counselors to better support couples with both economic and relationship difficulties.

The disrupted global economy due to the COVID‐19 pandemic resulted in economic hardship for many families. This economic downturn serves as a unique backdrop for family researchers because COVID‐19 brought both economic hardship and noneconomic changes in family life such as forced family time at home due to social distancing (Brock & Laifer, 2020). The COVID‐led economic crisis in South Korea is an interesting context to examine the impact of economic hardship on family relationships. Korea has a work‐oriented culture with one of the longest working hours in the world (OECD, 2021), and gatherings with coworkers are common after work. This culture had to change during the pandemic as more people worked remotely and returned home early to prevent COVID‐19 infections (J. Lee et al., 2020). This study extends the literature on the impact of economic hardship on couple relationships, which has often been drawn from the family stress model (FSM; Conger et al., 1994; Conger et al., 2010). We investigate the pathways from economic hardship to couple conflict during COVID‐19 and examine whether these pathways differ between Koreans with lower and higher socioeconomic status (SES).

## The FSM of couple relationships during COVID‐19

FSM (Conger et al., 1990; Conger et al., 1994) has been one of the leading conceptual models over the past 30 years in explaining the pathways from economic hardship to couple relationships. It theorizes on the indirect impact of economic hardship on couple dynamics through economic pressure and emotional distress. The couple‐level FSM (Conger et al., 1994; Conger et al., 2010) posits that economic hardship increases economic pressure, which, in turn, leads to emotional distress and eventually to greater challenges in couple functioning and relationships. The couple‐level FSM has been extended to child‐level outcomes through parental distress, interparental conflict, and negative parenting practices (Conger et al., 1992; Conger et al., 2010). Conger et al. (2010) named the couple‐level model “the FSM of romantic relationships” (p. 689) and the extended model the “extension of the FSM to lives of children” (p. 692). In this study, we focus on the couple‐level FSM.

The COVID‐related economic crisis provides a unique opportunity to study couple dynamics based on the FSM because economic hardship occurred along with forced couple proximity at home due to social distancing restrictions. In the nonempirical literature that emerged during COVID‐19, the FSM has often been mentioned while discussing how economic adversity because of the pandemic has led to difficulties and problems in intimate partnerships (Prime et al., 2020). However, to the best of our knowledge, empirical research drawn from the FSM has not been published in the context of COVID‐19 except for Low and Mounts's (2022) study. It is essential to accumulate empirical evidence examining the underlying processes from economic hardship to couple relationships during the economic downturn due to COVID‐19.

Since its development during the U.S. Midwest farm economic crisis in the 1980s, the FSM has been widely applied to empirical research in several countries, particularly during national and local economic downturns. The global applications of the FSM include, but are not limited to Korea (Kwon et al., 2003), China (Zhang et al., 2020), Argentina (Falconier, 2010), Belgium (Ponnet et al., 2016), Finland (Kinnunen & Feldt, 2004), Portugal (Fonseca et al., 2021), and Turkey (Aytaç & Rankin, 2009). However, only one to three studies were conducted on each of these non‐U.S. countries, which prevents us from making country‐specific generalizations or cross‐country comparisons (Falconier & Jackson, 2020). As for Korea, Kwon et al.’s (2003) well‐cited study is the only FSM study published in English, which was done in the context of the 1997 Korean economic crisis.

## Markers of economic hardship during COVID‐19

Economic hardship in the FSM refers to negative economic conditions such as low income and a high debt‐to‐asset ratio and negative financial events such as income loss and work instability (Conger & Conger, 2002; Conger et al., 2010). In a crushed economy, economic hardship may include negative economic *changes* at the family level because of economic declines (Conger et al., 1994). During the global economic downturn due to COVID‐19, families experienced economic hardship in the form of increased household debt, job loss, and reduced work hours (Center for Labor Trends Analysis, 2021; Chin et al., 2020; Y.‐K. Lee, 2021).

Despite the frequent use of the FSM in the literature, little attention has been paid to the specific markers of economic hardship. Instead, it has been common to use low income (C.‐Y. S. Lee et al., 2011; Ponnet et al., 2016), income‐to‐needs (Conger et al., 1990; Neppl et al., 2016), and debt (J. M. Park et al., 2017) as single proxies of economic hardship or to aggregate the multiple types of economic hardship as a latent variable (Kwon et al., 2003; Simons et al., 2016; Zhang et al., 2020). However, different markers of economic hardship may have distinct associations with couple relationships. A few exceptional studies in the FSM literature have examined the independent effects of debt and assets (Dew, 2007), debt and income (Fonseca et al. (2021), and income and length of unemployment (Kinnunen & Feldt, 2004).

In Korea, the amount of household debt rapidly increased during COVID‐19 (Chung, 2020), and previous studies have reported that household debt is more closely related to couple relationships compared to income or assets (Dew, 2007; Maeng & Han, 2019). The literature has shown inconsistent results on the link between household debt and couple relationships. Based on the FSM, Dew (2007) found both direct and indirect effects of consumer debt and marital conflict using a U.S. sample. However, in a Korean study, neither the amount of household debt nor the debt‐to‐income ratio had a relationship with marital instability (Chang & Lee, 2006). Among Portuguese couples, the indirect effect of family debt on family functioning and psychological well‐being was negative through economic pressure, but the direct effect of debt was positive (Fonseca et al., 2021). These mixed results imply that the amount or proportion of debt may not be an ideal marker of economic hardship because the impact of debt on couples may vary based on the types of debt and the reasons for borrowing (Chang & Lee, 2006; Dew, 2007). Thus, if detailed information on debt is not available, an increase in household debt may be an adequate variable reflecting economic hardship during COVID‐19 because additional borrowing may indicate economic disruptions during an economic downturn.

Job loss is another important marker of economic hardship that may increase economic pressure and emotional distress (Howe et al., 2004; Olesen et al., 2013). Like many other countries during COVID‐19, job loss was prevalent in Korea. The number of unemployed individuals was the highest during the pandemic since the 1997 Korean economic crisis (Kang et al., 2021). Studies have shown that job loss has adverse impacts not only on those who leave a job but also on their partners (Howe al., 2004; Moen et al., 2001).

Work hours generally decreased during the pandemic (Collins et al., 2021). The average work hours in Korea dropped by 4.1% between January and April 2020 (C. Park & Yoo, 2020). Reasons for fewer hours or less paid work during the pandemic may be that workplaces closed down in order to comply with social distancing orders, the shrunken labor market as a result of the economic decline, and care responsibilities because of closed schools and care facilities. Reduced work hours is a reasonable marker of economic hardship during COVID‐19 because working for less paid hours is likely to lead to lower income during an economic downturn.

Different markers of economic hardship during the pandemic, such as an increase in household debt, job loss, and a decrease in work hours, may have had different relationships with economic strain, emotional distress, and couple conflict. For example, household debt may have predated the pandemic while job loss and decreased work hours may be attributed directly to the pandemic. Although no or less paid work is expected to be negative for family finances, losing or reducing paid work during COVID‐19 also often meant forced close proximity for couples because of the stay‐at‐home restrictions. Therefore, the pathways from job loss and decreased work hours during COVID‐19 to couple relationships may be rather complicated.

Scholars tend to agree that extended time at home during COVID‐19 has been good for some couples but harmful for others (Günther‐Bel et al., 2020). On the one hand, job loss and reduced work hours are likely to lead to greater couple conflict because economic instability and abrupt changes in work arrangements negatively impact couples (Hill et al., 2017). Especially during the pandemic, given that partners may have already been worried about COVID‐19 infection and overwhelmed by the extended quarantine, no or less paid work may have been a double jeopardy not only for family finances but also for emotional distress and couple functioning. On the other hand, although no or less paid work tends to increase economic pressure, staying at home longer may not have been detrimental to emotional distress and couple conflict for some individuals. They may have found that the extended time spent together during COVID‐19 helped them build intimacy and better understanding (Günther‐Bel et al., 2020).

## Economic strain, emotional distress, and couple relationships during COVID‐19

Economic strain or pressure as a result of economic hardship is the second component of the FSM. This component is included in the FSM to reflect the psychological meaning of objective economic hardship (Conger & Conger, 2002; Conger et al., 2002; Conger et al., 2010). However, scholars have not agreed on the definition of this component. One strand of researchers, including those who developed the FSM, has defined economic pressure as tangible events such as “unmet material needs,” “the inability to pay bills or make ends meet,” and “having to cut back on even necessary expenses” (Conger et al., 2010, p. 690). However, the operational difference between economic hardship and pressure in the FSM is sometimes blurred, which may explain why several prior studies have chosen either economic hardship (C.‐Y. S. Lee et al., 2011; Simons et al., 2016; Simons & Steele, 2020) or pressure (Conger et al., 1994; Falconier, 2010; Hraba et al., 2000; Kwon et al., 2003; Robila & Krishnakumar, 2005; R. M. B. White et al., 2015) instead of both.

The second strand of researchers has defined economic pressure or strain as the subjective appraisal of economic circumstances (Falconier, 2010; Falconier & Jackson, 2020; Gudmunson et al., 2007; Kinnunen & Feldt, 2004; L. White & Rogers, 2000). This is based on stress theories like the stress process model (Pearlin et al., 1981) and family stress theories (Boss, 2002). The former distinguishes the subjective, secondary stressors from the objective, primary stressors, and the latter highlights both objective stressful events or conditions and the subjective perceptions of the objective changes. We take the second approach and utilize the term *economic strain* instead of *economic pressure* used in the original FSM. In this study, *increased economic strain* refers to the perceived level of negative changes in economic circumstances during COVID‐19 compared to pre–COVID‐19 circumstances.

Emotional distress has been reported as a key mediator in the pathways from economic hardship and strain to couple outcomes in the FSM (Conger et al., 2010). During COVID‐19, economic adversity elevated emotional distress (Low & Mounts, 2022; Prime et al., 2020). However, emotional distress was not directly related to marital problems for Korean and Turkish men in some FSM studies (Aytaç & Rankin, 2009; Kwon et al., 2003). These authors attributed this result to Korean and Turkish cultures where men can relieve their distress through social outlets such as spending time with their coworkers or friends outside the home. Assuming that this interpretation is accurate, it would be interesting to examine the relationship between emotional distress and couple conflict among Koreans during COVID‐19 as their social outlets were restricted because of social distancing measures.

Couple relationships is an outcome in the couple‐level FSM (Conger et al., 2010). The dimensions of the construct of couple relationships include negative interactions (e.g., conflict, hostility), positive interactions (e.g., warmth, support), and outcomes (e.g., satisfaction, adjustment, and stability; Falconier & Jackson, 2020). Although all of these dimensions have been used in FSM‐based studies, considerable attention has been paid to negative interactions because of the harmful nature of economic hardship (Gudmunson et al., 2007; Zhang et al., 2020). For instance, prior research has shown that economic strain or pressure is associated with couple conflict (Conger et al., 2002; Kwon et al., 2003; Ponnet et al., 2016; Robila & Krishnakumar, 2005). We also focus on couple conflict in this study as a representation of negative couple interactions during COVID‐19.

Couple conflict can be operationalized either as the level of negative interactions (Whitton et al., 2007) or the level of disagreement and tension over multiple domains of life (Papp, 2018). The latter has an advantage in the context of COVID‐19 because couples needed to adapt to the drastic changes that the pandemic brought in almost all domains of everyday life (J. Lee et al., 2020). Couples facing economic adversity may need to make more adjustments and, as a result, may experience greater conflict in these domains.

## Potential differences based on SES

It has been well documented that lower SES or lower income families are vulnerable to the negative impact of economic strain on couple relationships (see Falconier & Jackson, 2020, for a meta‐analysis). Studies have also found that economic instability during a recession creates greater challenges for families with limited income, insecure employment, and small business owners (Cho, 2017; L. White & Rogers, 2000). The same has been true during COVID‐19 (Prime et al., 2020). Lower SES families are more likely to encounter severe financial adversity during an economic crisis but also have limited economic resources to buffer the economic disruption. However, it is also true that families across the SES spectrum experience economic declines in a crushed economy (Schenck‐Fontaine & Panico, 2019). During COVID‐19, both lower and higher SES couples faced economic stress due to the massive impact of the pandemic on the global economy even though lower SES couples were more vulnerable to these challenges.

Most FSM studies have treated lower SES or income as a marker of economic hardship (Kinnunen & Feldt, 2004; C.‐Y. S. Lee et al., 2011; Ponnet et al., 2016) or have exclusively focused on low‐income families (Mistry et al., 2008). These previous approaches are limited because SES differences are largely missing in scholarly attempts to understand the complex pathways from economic hardship to family outcomes. As an exception, Fonseca et al.’s (2021) study of Portuguese couples found that the association between family income and economic pressure was stronger for higher SES groups, whereas the negative link between economic pressure and family functioning was stronger for lower SES men. In the context of Korea, two studies have reported the moderating role of income and assets in the relationship between debt and family functioning (Maeng & Han, 2019; J. M. Park et al., 2017). These studies suggest that SES can be a potential moderator in the pathways from economic hardship to couple conflict. In the present study, we aim to examine the differences between lower and higher SES groups in the process of economic stress.

## Current study

We test a path model that posits associations among economic hardship, increased economic strain, emotional distress, and couple conflict among married Koreans during the pandemic. We hypothesize that when couples accumulated more debt, could not work for pay, or worked for fewer hours during COVID‐19, they would perceive an elevated level of economic strain because of a decline in their overall family finances. This heightened economic strain would lead to higher levels of perceived stress and, in turn, couple conflict as suggested in the FSM. We examine whether these pathways differ between lower and higher SES groups.

**FIGURE 1:**
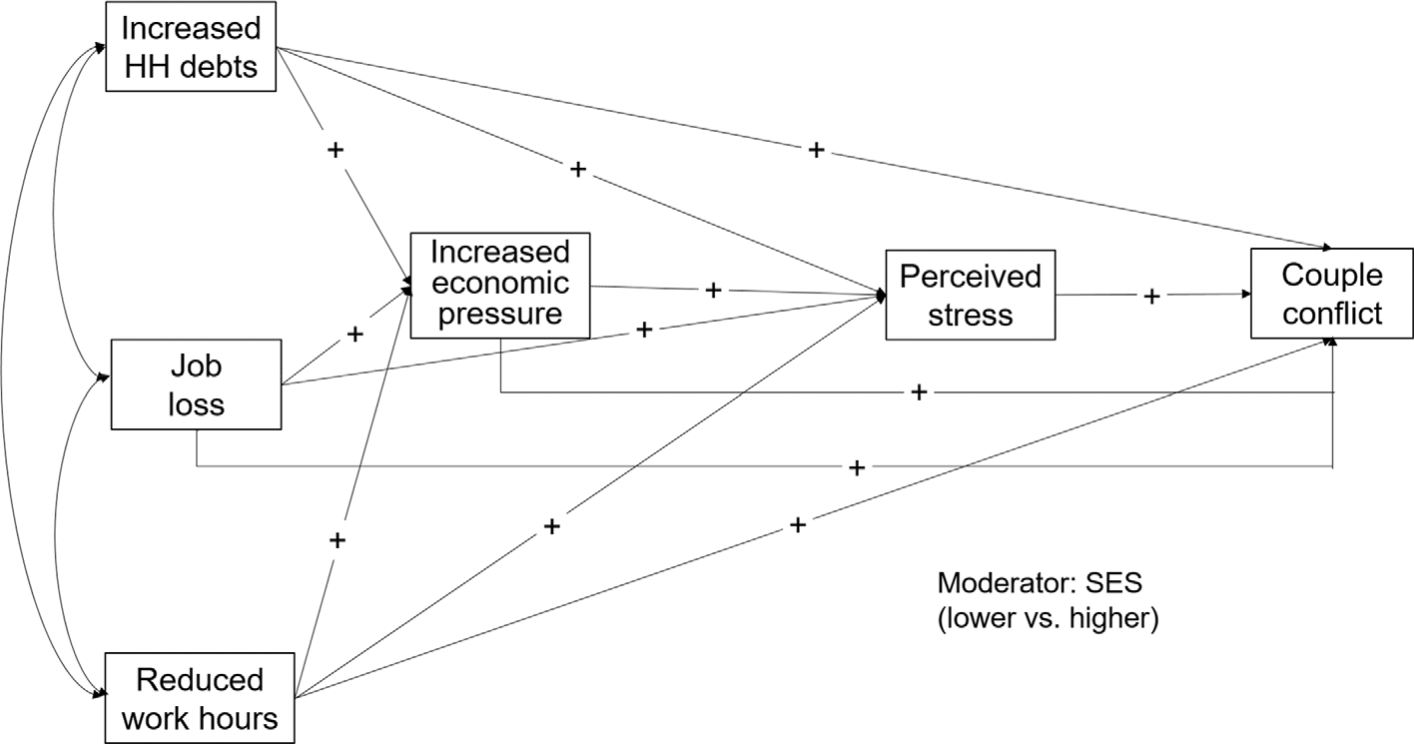
Conceptual model *Note*. HH = household; SES = socioeconomic status

Figure 1 displays our conceptual path model. Our conceptual model is largely drawn from the FSM of romantic relationships (Conger et al., 2010) with a few modifications. In the first modification, we hypothesize all the possible paths between the study variables to examine both the direct and indirect effects. This approach is unlike the FSM (Conger et al., 2010), which posits only indirect associations between economic hardship and couple relationships. Our focus on the direct links is based on the literature that has shown the direct effect of economic hardship markers on marital relationships (Dew, 2007; Kinnunen & Feldt, 2004; Kinnunen & Pulkkinen, 1998) and family functioning (Fonseca et al., 2021). Direct effects of economic pressure have also been found on marital conflict and problems in multiple countries (Aytaç & Rankin, 2009; Hraba et al., 2000; Kwon et al., 2003; Ponnet et al., 2016). Some markers of economic hardship have also been found to have a direct link to emotional distress (Kinnunen & Pulkkinen, 1998; Neppl et al., 2016). In the context of COVID‐19, it is plausible to hypothesize that economic hardship and strain have direct associations with couple conflict because financial adversity may have immediately led to disagreements and tensions for couples who had to adapt to the unprecedented and drastic changes that the pandemic brought to their daily lives. The literature has also noted the acute impact of economic hardship and pressure on couples during macroeconomic downturns (Fonseca et al., 2021; Fonseca et al., 2016; Kwon et al., 2003).

The second modification is our interest in negative *changes* in economic hardship and strain during COVID‐19 instead of the actual level. Because economic conditions and stress can change in a crushed economy regardless of SES, the changes that occurred after the pandemic began is an adequate focus when comparing the impact of economic hardship between lower and higher SES individuals. Further, we do not use the actual levels of economic hardship and strain because they would be highly correlated with our moderator, SES.

Lastly, due to the unavailability in the data, our model does not include the relationship quality or stability variable, the final outcome in the FSM of romantic relationships (Conger et al., 2010). Although it would be ideal to test all constructs in the FSM, this study contributes to the literature by carefully considering the complexity of the FSM of romantic relationships.

## METHOD

### Data collection and participants

We used a subset of data from a larger research project called the “Impact of COVID‐19 on Korean Families,” on which the authors of this study collaborated. The data were collected from 1,055 Korean adults aged 20 to 64 years from May 19–25, 2020, using an online survey to comply with the social distancing guidelines. The first surge of COVID‐19 cases was in the city of Daegu and adjacent areas in February and March, 2020. In mid‐May, when the data were collected, Koreans in the Seoul metropolitan area witnessed the first surge in the number of confirmed cases. The total number of confirmed cases also increased throughout the nation in mid‐May. A leading survey company in Korea recruited participants from a substantial number of online survey panels with diverse potential respondents across the country. Quota sampling was used based on the proportions of the national population in terms of age, gender, and region (Seoul, Gyeonggi‐Gangwon, Chungcheong, Honam, and Yeongnam). Although some variables in the data set were measured using single items, this data set is valuable given the timing of data collection and the wide spectrum of family‐related variables.

For the present study, we selected 605 respondents who self‐identified as married and lived with their partners at the time of data collection (282 women and 323 men). Although it is important to consider other nonmarital relationships such as cohabitation, our data did not provide information on nonmarital partnerships because it is not easy to identify cohabiting couples using a survey in Korea due to the stigma around it. Our sample included married Koreans aged 22 and 64 years (*M* = 48.9 years, *SD* = 9.34). Of these, 67.6% had at least some college education, and 52.2% were from dual‐income families. The average duration of their marriage was 19.5 years (*SD* = 11.2), and the average number of children was 1.6 (*SD* = 0.79). We did not have couples in the data but instead selected individuals in a married relationship.

### Measures

#### Economic hardship and strain

Economic hardship was assessed by asking whether respondents or their partner experienced an increase in household debt, job loss, or a reduction in work hours during the COVID‐19 pandemic. For increased household debt, we asked the following question: “How has your household debt changed during COVID‐19 compared to mid‐January 2020?” A 5‐point Likert scale was used (1 = *greatly decreased*, 2 = *slightly decreased*, 3 = *hasn't changed much*, 4 = *slightly increased*, 5 = *greatly increased*). Job loss was coded 1 if the respondent or their partner had not worked for pay because of the COVID‐19 pandemic for the preceding 3 months (mid‐February to mid‐May 2020) and 0 otherwise. For reduced work hours, we asked the participants to report the number of weekly hours that the respondent and their partner had worked before (mid‐January) and during (mid‐April) the COVID‐19 pandemic. Based on these responses, we created a variable of reduced work hours (1 = either spouse worked for fewer hours during COVID‐19).

Increased economic strain was measured by the subjective appraisal of changes in overall family economic conditions during the pandemic. The respondents were asked, “How has your family's economic situation changed during COVID‐19?” (1 = *greatly improved*, 2 = *slightly improved*, 3 = *hasn't changed much*, 4 = *slightly worsened*, 5 = *greatly worsened*). Thus, a higher score indicates a greater decline in the family's overall economic circumstances, which is interpreted as a higher level of increased economic strain during the pandemic in this study.

#### Perceived stress

Our emotional distress variable was perceived stress measured by the Korean version of Cohen et al.’s (1983) Perceived Stress Scale (Yoon & Kim, 2019). In the FSM literature, a wide range of variables have been used as indicators of emotional distress such as depressive symptoms, anxiety, anger, and hostility (Conger et al., 2010; Neppl et al., 2016). Perceived stress is a reasonable indicator of emotional distress for nonclinical samples (Yan et al., 2021) although the use of perceived stress is limited in the FSM literature. The respondents were asked how often they experienced the listed symptoms of stress in the preceding 3 months (from mid‐February to mid‐May 2020). The original scale in English included 14 items, but we used the Korean version, which was validated with 10 items (Yoon & Kim, 2019). Sample items include “How often have you been upset because of something that happened unexpectedly?” and “How often have you felt nervous and stressed?” on a 5‐point Likert scale (1 = *never*, 5 = *very often*), with higher scores indicating higher levels of emotional distress. We used a mean score, and Cronbach's alpha was .81.

#### Couple conflict

The respondents were asked how often they experienced conflict with their partners during COVID‐19 in eight aspects: (a) relationships with parents and siblings, (b) relationships with their partner's parents and siblings, (c) lifestyle issues such as drinking and coming back home late at night, (d) child rearing and education, (e) economic issues, (f) division of housework and childcare, (g) sexual life, and (h) personality or ways of thinking. The response categories were *not at all* (1), *rarely* (2), *sometimes* (3), *often* (4), and *very often* (5). A not applicable (N/A) option was also provided for those who did not have living parents(−in‐law) and siblings(−in‐law) or those who did not have children. We used an arithmetic mean, with higher scores indicating higher levels of couple conflict. Cronbach's alpha was .90.

#### SES

We assessed SES subjectively by asking, “How would you evaluate your socioeconomic status considering income, occupation, education, and assets?” (1 = *low*, 2 = *lower middle*, 3 = *middle*, 4 = *upper middle*, 5 = *high*). For our multigroup analysis, the sample was divided into two groups: *lower* SES (39%) by aggregating responses of “low” (4.6%) and “lower middle” (34.4%) versus *higher* SES (61%) by aggregating “middle” (47.1%), “upper middle” (13.6%), and “high” (0.3%). We chose subjective SES because it is an effective measure of SES whereas the markers of objective SES are very difficult to define and combine into a single variable (Andersson, 2018). Particularly in Korea, education, income, and occupation status are interpreted differently depending on numerous factors including one's birth cohort.

### Analytical strategy

We conducted a multigroup path analysis using Mplus 8 to compare group differences between lower and higher SES across all hypothesized pathways in the proposed model. The significance of indirect effects was tested using bootstrapping procedures with 1,000 bootstraps and a bias‐corrected 95% confidence interval (CI). The indirect effect was considered significant when the 95% CI did not include zero. We controlled for the effects of the respondent's gender and age on all exogenous and endogenous variables in the proposed path model.

## RESULTS

Table 1 shows the descriptive statistics and correlations among the study variables for *lower* and *higher* SES groups along with *t*‐test results comparing these two groups. As for household debt, 26.2% of the *lower* SES group and 13.5% of the *higher* SES group reported an increase in household debt. Job loss due to the pandemic was reported for 13.1% of *lower* SES respondents and 11.4% of *higher* SES individuals. A decrease in work hours during COVID‐19 was more common in the *lower* (29.2%) than in the *higher* (18.7%) SES group. On average, the extent of increase in economic strain during COVID‐19 was greater in the *lower* SES group than in the *higher* SES group (*M*_lower_ = 3.56, *M*_higher_ = 3.33, *t* = 4.54, *p* < .000). The *lower* SES group reported higher levels of perceived stress (*M*_lower_ = 2.93, *M*_higher_ = 2.74, *t* = 4.69, *p* < .000) and couple conflict (*M*_lower_ = 2.33, *M*_higher_ = 2.17, *t* = 2.62, *p* < .01) than the *higher* SES group.

**TABLE 1:** Intercorrelations and descriptive statistics for lower and higher SES groups

Variable (range)	1	2	3	4	5	6	7	8
1. Increased HH debt (1–5)	—	.11*	.16**	.34***	.15**	.16**	−.01	−.03
2. Job loss (1 = *yes*)	.11	—	.03	.30***	.22***	.19***	.14**	.02
3. Reduced work hours (1 = *yes*)	.03	−.06	—	.37***	.15**	.18***	.04	−.03
4. Economic strain (1–5)	.31***	.20**	.36***	—	.26***	.21***	.03	.05
5. Perceived stress (1–5)	.27***	.13*	.10	.28***	—	.41***	.06	−.14**
6. Couple conflict (1–8)	.30***	.13*	.06	.31***	.39***	—	−.07	.02
7. Female	.10	.08	−.04	.12	.13	.07	—	−.04
8. Age (20–64 years)	−.10	.12	−.05	.02	−.17*	−.00	−.03	—
	*M* (*SD*)	%	%	*M* (*SD*)	*M* (*SD*)	*M* (*SD*)	%	*M* (*SD*)
Full sample (*N* = 605)	3.15 (.60)	12.1	22.8	3.42 (.63)	2.81 (.50)	2.23 (.74)	46.6	48.9 (9.34)
Lower SES (*n* = 236)	3.23 (.68)	13.1	29.2	3.56 (.66)	2.93 (.47)	2.33 (.71)	44.9	50.2 (9.20)
Higher SES (*n* = 369)	3.10 (.54)	11.4	18.7	3.33 (.60)	2.74 (.50)	2.17 (.75)	47.7	48.1 (9.36)
*t*	2.72**	—	—	4.54***	4.69***	2.62**	—	2.61**

*Note*: HH = household; SES = socioeconomic status. Correlations for the *lower* SES sample are below the diagonal. Correlations for the *higher* SES sample are above the diagonal.

* *p* < .05.

** *p* < .01.

*** *p* < .001.

**FIGURE 2:**
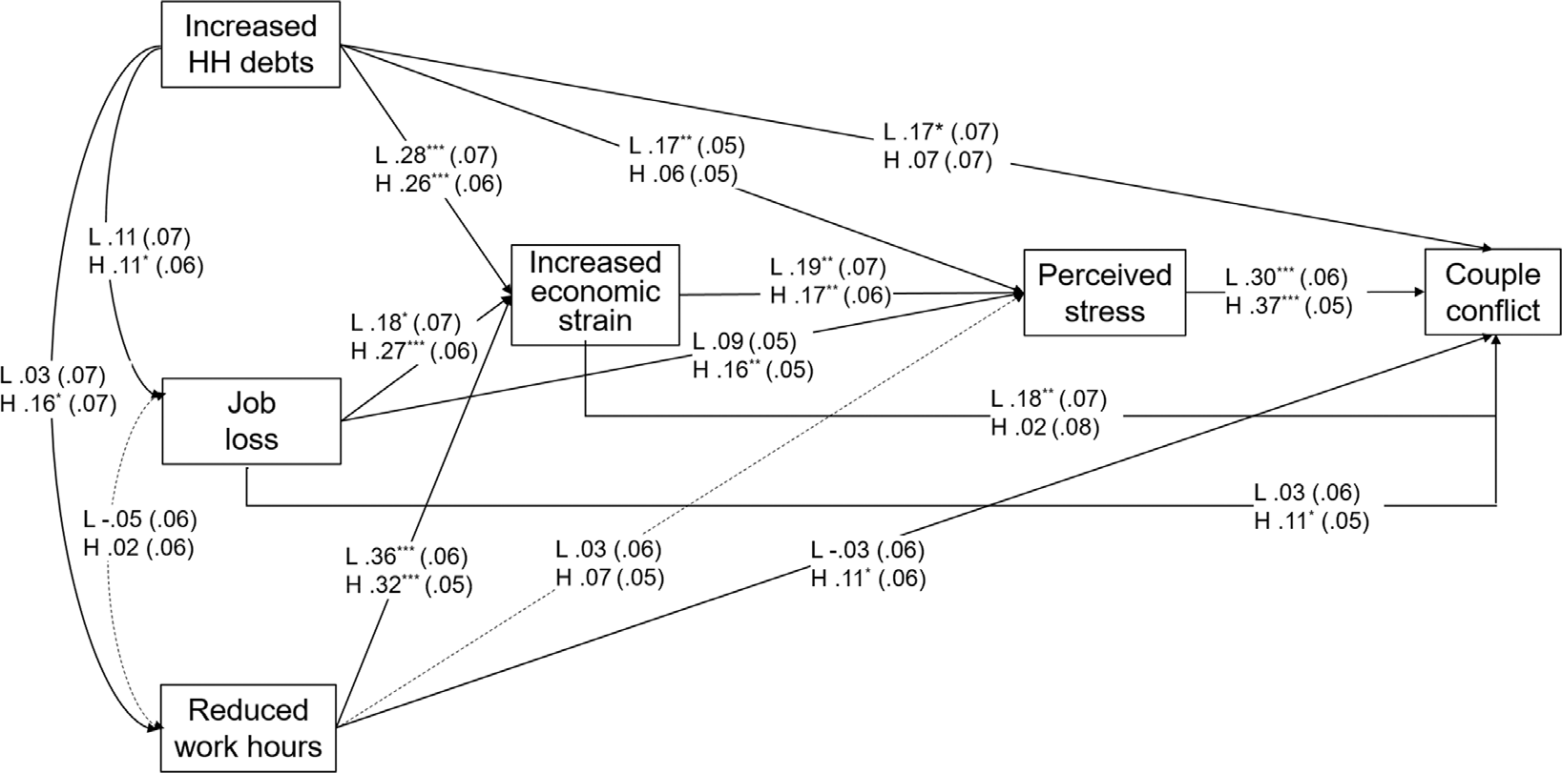
Multigroup standardized path coefficients of and standard errors in the proposed model *Note*. H = higher SES; HH = household; L = lower SES; SES = socioeconomic status. Standard errors are in parentheses. Controlling for gender and age not shown. Dashed lines indicate that the paths for both lower and higher SES groups are insignificant. *p < .05. **p < .01. ***p < .001

Figure 2 shows the results of our multigroup path analysis, which compared the paths of our proposed model between *lower* and *higher* SES groups after controlling for age and gender. For direct relationships between the three markers of economic hardship and couple conflict, increased household debt had a direct link with couple conflict only in the *lower* SES group (β_lower_ = .17, *p <* .05). Job loss (β_higher_ = .11, *p <* .05) and reduced work hours (β_higher_ = .11, *p <* .05) were directly associated with couple conflict in the *higher* SES group alone.

All three markers of economic hardship were indirectly associated with couple conflict sequentially through increased economic strain and perceived stress in both SES groups. Specifically, increased household debt (β_lower_ = .28, β_higher_ = .26, both *p*s < .001), job loss (β_lower_ = .18, *p <* .05; β_higher_ = .27, *p <* .001), and reduced work hours (β_lower_ = .36, β_higher_ = .32, both *p*s < .001) were all linked to an increase in subjective economic strain. In both SES groups, heightened economic strain was related to a higher level of perceived stress (β_lower_ = .19, β_higher_ = .17, both *p*s < .01). Perceived stress was significantly associated with more frequent couple conflict (β_lower_ = .30, β_higher_ = .37, both *p*s < .001). That is, when the sequential mediation of increased economic strain and perceived stress was considered, all three markers of economic hardship were indirectly related to couple conflict in both SES groups.

Some group differences were found for the indirect pathways with a single mediator. In the *lower* SES group, an increase in household debt was directly associated with perceived stress (β_lower_ = .17, *p <* .01) whereas in the *higher* SES group, job loss had a direct relationship with perceived stress (β_higher_ = .16, *p <* .01). That is, when perceived stress was the sole mediator, an increase in household debt in the *lower* SES group and job loss in the *higher* SES group were indirectly associated with more frequent couple conflict, even without intensified economic strain. Finally, in the *lower* SES group, heightened economic strain was directly related to more frequent couple conflict (β_lower_ = .18, *p <* .01). This result indicates that, in the *lower* SES group, an increase in economic strain followed by economic hardship was directly linked to couple conflict without the mediating role of perceived stress at the individual level.

Table 2 displays the significance and strength of the total indirect and grand total (direct + total indirect) effects of all proposed pathways for the *lower* and *higher* SES groups. First, for the *lower* SES group, the effect of increased household debt on couple conflict was the strongest among the three economic hardship markers both for the total indirect (β_lower_ = .12, 95% CI [.06, .19]) and the grand total (β_lower_ = .29, CI [.16, .42]) effects. In contrast, in the *higher* SES group, the association from increased debt to couple conflict was not significant both for the total indirect effect (β_higher_ = .04, CI [−.01, .10]) and the grand total effect (β_higher_ = .12, CI [−.01, .23]). Second, the total indirect effect of job loss on couple conflict was significant for both the *lower* (β_lower_ = .07, CI [.02, .12]) and *higher* (β_higher_ = .08, CI [.02, .14]) SES groups, but the grand total effect was significant only for the *higher* SES group (β_higher_ = .19, CI [.09, .27]). Finally, for reduced work hours, the grand total of direct and indirect effects was significant only for the *higher* SES group (β_higher_ = .16, CI [.06, .26]) even though the total indirect effect was significant only for the *lower* SES group (β_lower_ = .09, CI [.03, .17]). This may be because of the nonsignificant but negative indirect relationship between reduced work hours and couple conflict for the *lower* SES group.

**TABLE 2:** Standardized bootstrap estimates and bias‐corrected 95% confidence intervals for indirect and total effects (*N* = 605)

			BC 95% CI	
Effect	β	*SE*	*LL*	*UL*
Lower SES (*n* = 236)				
Increased HH debts → Couple conflict				
Total indirect	.12***	.03	.06	.19
Total: Indirect + direct	.29***	.07	.16	.42
Job loss → Couple conflict				
Total indirect	.07**	.03	.02	.12
Total: Indirect + direct	.10	.06	−.03	.22
Reduced work hours → Couple conflict				
Total indirect	.09**	.04	.03	.17
Total: Indirect + direct	.06	.06	−.05	.17
Higher SES (*n* = 369)				
Increased HH debt → Couple conflict				
Total indirect	.04	.03	−.01	.10
Total: Indirect + direct	.12	.06	−.01	.23
Job loss → Couple conflict				
Total indirect	.08**	.03	.02	.14
Total: Indirect + direct	.19***	.05	.09	.27
Reduced work hours → Couple conflict				
Total indirect	.05	.03	−.01	.12
Total: Indirect + direct	.16***	.05	.06	.26

*Note*: BC = bias corrected; CI = confidence interval; HH = household; *LL* = lower limit; SES = socioeconomic status; *UL* = upper limit.

* *p* < .05.

** *p* < .01.

*** *p* < .001.

## DISCUSSION

In the context of the global economic downturn brought on by COVID‐19, we investigated the pathways from economic hardship to couple conflict through increased economic strain and perceived stress among married Koreans. With special attention to three markers of economic hardship (increased household debt, job loss, and reduced work hours), we found that the process of economic stress differed between the *lower* and *higher* SES groups. This study contributes to the literature by providing timely empirical evidence for the mechanism from economic adversity to couple relationships during COVID‐19 based on the FSM. Our focus on SES differences in the mechanism is innovative in the FSM literature, which has mostly considered low income or SES as an indicator of economic hardship rather than as a moderator.

Overall, our results supported the FSM of romantic relationships (Conger et al., 2010), which hypothesizes indirect pathways from economic hardship and couple relationships through economic pressure and emotional distress. We found that all our economic hardship markers were associated with elevated economic strain, which, in turn, was related to a higher level of perceived stress and then to more frequent couple conflict. These sequential associations were found for both *lower* and *higher* SES groups.

We also found direct links between increased household debt and couple conflict for the *lower* SES group. This is in line with Dew's (2007) findings using a U.S. sample, and suggests that more borrowing led to negative couple interactions both directly and indirectly among *lower* SES Koreans during COVID‐19. The direct impact of debt supports previous studies that have reported the acute threat of economic adversity on couple relationships during an economic crisis (Fonseca et al., 2016; Kwon et al., 2003). The direct association may be because an increase in household debt was related to greater marital disagreements on whether or not to borrow more, how much and from where they should borrow, and how to manage and repay the debt (Dew, 2007). Out of the three markers of economic hardship, the impact of increased debt was the most severe on couple conflict for the *lower* SES group based on the strongest grand total effect of increased debt. This implies that *lower* SES individuals are particularly vulnerable to couple conflict when they must incur more debt during an economic downturn, even without elevating economic strain and perceived stress.

For the *higher* SES group, however, the direct relationship between increased household debt and couple conflict was not significant. Like what the FSM postulates, for the *higher* SES group, increased debt only had an indirect impact on couple relationships through the sequential mediation of elevated economic strain and emotional distress. This nonsignificant direct association is similar to previous research, which has failed to find a direct link between debt and family dynamics (Chang & Lee, 2006). Although the *higher* SES group was not free from the sequential indirect effect of increased debt through elevated economic strain and perceived stress, greater borrowing was not a direct threat in this group because debt may have been related to accumulated assets such as a home and stocks or better cash flow in *higher* SES households (Chang & Lee, 2006; J. M. Park et al., 2017). This finding was unlike the *lower* SES group whose debt was more related to survival during a crushed economy. As *higher* SES couples had economic resources to repay debt, it seems that they could avoid the direct impact of increased debt.

Unlike increased household debt that had a direct impact on couple conflict only for *lower* SES Koreans, job loss and reduced work hours had a direct association with couple conflict only for the *higher* SES group. That is, the impact of job loss and reduced work hours on couple conflict was both direct and indirect among the *higher* SES group, but the impact was only indirect among the *lower* SES group. As it is very likely that *lower* SES individuals had already experienced unstable employment and changes in work arrangements before COVID‐19, unemployment and reduced work hours may not have been acute stressors. In contrast, job loss and a reduction in work hours because of COVID‐19 may have been unexpected with abrupt changes for *higher* SES couples, which directly created more frequent conflict.

We also found indirect links from economic hardship to couple conflict through perceived stress as a single mediator. Increased household debt was directly associated with a higher level of perceived stress only for the *lower* SES group, which, in turn, led to couple conflict. In contrast, job loss was directly related to perceived stress for the *higher* SES group only, which eventually led to more frequent couple conflict. Reduced work hours did not have a direct association with perceived stress for any SES group. These findings suggest that incurring greater debt and not working for pay were both economic and psychological stressors for married Koreans during COVID‐19. These direct effects of debts and unemployment on perceived stress may be the result of the level of uncertainty and concern that were already high because of the pandemic, and debt and unemployment raised their uncertainty and concern all the more. Our results also show that an increase in household debt for the *lower* SES group and unemployment for the *higher* SES group, respectively, were directly negative for emotional distress during the pandemic.

We found a direct relationship between heightened economic strain and couple conflict only for the *lower* SES group. Although this direct link was not hypothesized in the FSM, our result is in line with previous non‐Western studies that have reported a direct association between economic pressure, which is the psychological meaning of economic hardship, and marital problems among Korean couples (Kwon et al., 2003) and Turkish married individuals (Aytaç & Rankin, 2009). According to these authors, this direct effect is unique in non‐Western countries where traditional gender roles are strong. These authors inferred that economic pressure can be a direct threat to marriage because economic adversity is seen as the male partner's failure to fulfill the breadwinning role in these countries (Aytaç & Rankin, 2009; Kwon et al., 2003). It is interesting that the same direct effect was found in our study almost 20 years after Kwon et al.’s (2003) findings on marital conflict during the 1997 Korean economic crisis. Further research is necessary to verify whether the direct link between elevated economic strain and more frequent couple conflict is because of traditional gender roles.

Finally, we found a direct effect of perceived stress on couple conflict for both the *lower* and *higher* SES samples after controlling for gender and age. Although this effect is postulated in the FSM, Kwon et al. (2003) and Aytaç and Rankin (2009) did not find a direct association between emotional distress and marital problems for Korean and Turkish men. These authors speculated that this lack of a direct association was because of the long work hours and social outlets in Korea and Turkey, which prevented men from transmitting their emotional distress to their marriage. Assuming that this speculation is accurate, the direct link between perceived stress and couple conflict in this study can be understood in the context of COVID‐19. As social distancing measures forced Korean couples to stay together at home and to avoid social time outside, the intervening role of social networks would have been far weaker. A recent Korean study also reported that support from friends did not moderate the negative impact of a lower economic standing during the pandemic (Son et al., 2021).

## IMPLICATIONS AND LIMITATIONS

Family life educators, therapists, and policy makers should help couples prevent or reduce the negative impact of economic hardship on couple relationships, particularly in the context of a crisis like COVID‐19. Based on our results, which revealed differential pathways between the three markers of economic hardship and couple conflict by SES, we suggest that family practitioners should consider the distinct impact of increased debt, job loss, and reduced work hours on couple adjustment. They should also consider the couple's preexisting socioeconomic resources. Given the strong effect of increased household debt on couple conflict for the *lower* SES group, professionals can help couples with limited economic resources develop financial and psychological strategies to cope with household debt. Given that *higher* SES individuals were particularly vulnerable to job loss, it would be useful to address coping strategies pertaining to unemployment during an economic crisis while working with *higher* SES couples. Although family life education and therapy are widely practiced in Korea, family life educators and therapists are not adequately trained to deal with economic adversity because of their specialization and focus on relationship issues. Thus, it would be helpful if family practitioners collaborate with financial counselors and therapists to develop interventions to assist couples experiencing both economic and relationship difficulties (Falconier & Jackson, 2020). It is also essential for family life educators and therapists to help economically stressed couples locate available policy assistance given that various public policies and services have been implemented to lessen the negative impact of COVID‐19–related economic problems. Finally, given that perceived stress at an individual level mediated the association between economic hardship and couple conflict in this study, it would be helpful for family practitioners to lower each partner's distress in an effort to lessen the harmful effect of economic stress on couple functioning.

This study has a few limitations given that it is the first empirical endeavor to test the FSM in the early phase of the pandemic. Despite our strength of focusing on changes during COVID‐19 and SES differences, we did not have dyadic data. Causal and reciprocal relationships among the study variables are unknown because of the cross‐sectional survey design. As the interactionist perspective on the FSM suggests (Conger et al., 2010), couple conflict as a result of economic stress during the early pandemic could lead to poorer economic circumstances and greater perceived stress in the later period of the pandemic. The strength of this interactional relationship may differ by SES. Our measurement of economic hardship variables could have been scrutinized further. For example, we did not assess the types of debt and jobs and did not consider the detailed reasons for unemployment or reduced work hours. Although reduced work hours was an appropriate hardship marker given the positive link between reduced work hours and increased economic strain in this study, working for less hours could have been a simple change of work environment rather than an economic hardship for some people due to the pandemic.

The present study is also restricted because gender and age were only control variables and parenting responsibilities were not considered. Gender plays an important role in the process of economic stress (Fonseca et al., 2016), and the impact of economic stress on couple conflict may differ by gender particularly during COVID‐19 when care and housework burdens were heightened due to social distancing restrictions. Future research should pay more attention to gender differences in the FSM during COVID‐19. In addition, this study was limited because we did not analyze differences across family development stages. Unlike most previous FSM research that has investigated couples with younger children or adolescents, our data came from couples in various family development stages. This diversity of our sample is a strength because couples in all stages can face economic hardship during an economic crisis, but paying less attention to developmental differences is a limitation. We suggest a more thorough investigation into family development stages or children's developmental phases for future research.

Despite these limitations, this study contributes to the literature by providing timely evidence of a complicated mechanism among economic hardship, elevated economic strain, perceived stress, and couple conflict during the economic crisis brought on by COVID‐19. Our results are a unique addition to the FSM literature by revealing both direct and indirect pathways, and by showing the distinct impact of increased household debt, job loss, and reduced work hours. We found that the FSM worked differently based on the marker of economic hardship. Finally, this study is meaningful as it documents the pathways from economic hardship to couple conflict as distinguished by SES, a factor that has not been examined as a moderator in the FSM literature.
